# Retinal Vessel Segmentation Algorithm Based on Residual Convolution Neural Network

**DOI:** 10.3389/fbioe.2021.786425

**Published:** 2021-12-10

**Authors:** Shuang Xu, Zhiqiang Chen, Weiyi Cao, Feng Zhang, Bo Tao

**Affiliations:** ^1^ Key Laboratory of Metallurgical Equipment and Control Technology, Ministry of Education, Wuhan University of Science and Technology, Wuhan, China; ^2^ Hubei Key Laboratory of Mechanical Transmission and Manufacturing Engineering, Wuhan University of Science and Technology, Wuhan, China; ^3^ Precision Manufacturing Institute, Wuhan University of Science and Technology, Wuhan, China

**Keywords:** retinal vessel segmentation, convolution neural network (CNN), residual network, fundus image, attentional mechanism, deep supervision

## Abstract

Retinal vessels are the only deep micro vessels that can be observed in human body, the accurate identification of which has great significance on the diagnosis of hypertension, diabetes and other diseases. To this end, a retinal vessel segmentation algorithm based on residual convolution neural network is proposed according to the characteristics of the retinal vessels on fundus images. Improved residual attention module and deep supervision module are utilized, in which the low-level and high-level feature graphs are joined to construct the encoder-decoder network structure, and atrous convolution is introduced to the pyramid pooling. The experiments result on the fundus image data set DRIVE and STARE show that this algorithm can obtain complete retinal vessel segmentation as well as connected vessel stems and terminals. The average accuracy on DRIVE and STARE reaches 95.90 and 96.88%, and the average specificity is 98.85 and 97.85%, which shows superior performance compared to other methods. This algorithm is verified feasible and effective for retinal vessel segmentation of fundus images and has the ability to detect more capillaries.

## 1 Introduction

The deep neural network is a typical bio-inspired intelligence computation technique, which based on the principles of biological processes that the connectivity pattern between neurons resembles the organization of the animal visual cortex. Due to the incredible abilities to solve complex problems, it has attracted much attention from many scholars and have been successful applied to solve complex real-world problems (Sun et al., 2020b; Sun et al., 2020c; Chen et al., 2021a; Jiang et al., 2021a). Retinal vascular occlusion, hypertensive arteriosclerosis and diabetic retinopathy are the most common diseases in retinal diseases and also the main cause of blinding in the world (Horton et al., 2016). It is estimated that the number of people with vision loss will double by 2050 (Varma et al., 2016). Early detection and treatment can preserve 90% vision and also help the auxiliary medical management departments to formulate preventive measures to reduce the number of newly diagnosed cases and reduce the medical related economic burden (Das, 2016; Guo et al., 2018; Sun et al., 2020a). The morphological structure of retinal vessels has important reference value for the diagnosis of the diseases. Accurate and rapid segmentation of retinal vessels is necessary for the treatment. However, the segmentation requires manual labeling by experts at present, which is not only time-consuming and laborious, but also not accurate enough to carry out large-scale labeling and segmentation. It is significant to develop retinal vessel segmentation algorithms to improve the intelligence of computers in the aspect of disease diagnosis and health screening. Due to the excellent performance of bio-inspired computation method (Deng et al., 2020; Zhao et al., 2020; Tao et al., 2021a; Jiang et al., 2021b), researchers tried various types of algorithms to improve retinal vessel recognition, focusing on the segmentation and extraction of detailed information of connected vessel stems and terminals.

In addition, retina is one of the most reliable, stable and hard-forged information among all the biological features used for identification. As early as the 1930s, some foreign scholars proposed the unique theory of the distribution of retinal vessels. Subsequent studies have shown that the distribution of retinal vessels is different, even for twins (Cao et al., 2017; Zhang 2020). Aside from changes in retinal features due to trauma and disease, the shape of the retinal vessels remains stable throughout life, making it ideal for identification. In the foreseeable future, retina recognition technology has a great hope to be applied to online payment, access control, automatic withdrawal and other civil fields with high security requirements. Therefore, the research on retina recognition technology has great value and good prospects.

In this paper, we proposed a novel retinal vessel segmentation algorithm based on residual convolution neural network, which involves three major steps: 1) the residual learning is introduced in the network structure; 2) the atrous spatial pyramid pooling is built to learn the feature information of different receptive fields; 3) the residual attention module and deep supervision module are applied to improve the accuracy of identifying the capillaries.

The main contribution of this work is to propose the novel algorithm for segmenting retinal vessel from fundus images. It outperforms many recent works, including several methods using deep learning. The proposed algorithm can obtain complete retinal vessel segmentation, including connected vessel stems and terminals, especially the capillaries, and is fast and easily scalable to any fundus image size. Three more specific contributions are also worth mentioning. Firstly, an improved residual attention module is built and combined with a designed deep supervision module, that successfully solves the problem of gradient disappearance and gradient explosion caused by the depth of convolutional neural network. Secondly, an encoder-decoder network structure is constructed, in which the low-level and high-level feature graphs are joined together. It effectively avoids inefficient learning and sharing in training. Thirdly, atrous spatial pyramid pooling is constructed by introducing the atrous convolution, that effectively enlarges the receptive field while reducing the number of training parameters. Our proposed algorithm can be used to help doctors diagnose retinal disease, and could also support future computer-assisted diagnosis, health screening, and retina identification.

The rest of this paper is organized as follows: *Related Work* discusses the related work contributed by researchers, followed by the *Network Structure*, in which each block has been described in detail. *Experiment and analysis* indicate the data acquisition, experimental results, comparison and analysis. *Conclusion and future work* conclude the paper with a summary and future research directions.

## 2 Related Work

Retina is made up of complex blood vessels surrounding the tiny nervous system at the back of the eyeball, which contains a large number of features. Researchers have proposed many retinal vessel segmentation algorithms over the years, including matching filtering method (Singh and Srivastava, 2016; Roy et al., 2019), vascular tracking method (Pal et al., 2019; Alaguselvi and Murugan, 2021), image morphology processing and deep learning method (Grewal et al., 2018; Soomro et al., 2019).

Traditional image segmentation methods, as matching filtering method and vascular tracking method, focus on the various filters design and the image morphology process to achieve the purpose of retinal vessel segmentation (Li G. et al., 2019; Li J. et al., 2021). In the reference (Pachade et al., 2020), a unique combination of morphological operations, background estimation, and iterative thresholds was applied to achieve the retinal vessel segmentation. Girard et al. (2019) defined branches by nodes and combined with graph propagation to do the segmentation and classification, and Li et al., 2020b used a deep forest-based segmentation algorithm for retinal vessels. However, in some lesion areas and optic disc edges, these methods may incorrectly detect points as blood vessels. Fan et al. (2019) combined the matched filters and morphological process based on different Gaussian filters in different directions and vector field divergence. In the meantime, multi-scale wavelet transforms (Tian et al., 2021) was used to fuse feature images, and the maximum value of each pixel was calculated to obtain retinal vessel detection images. But the interference of optic disc will lead to the degradation of segmentation performance. Dharmawan et al., 2019a designed a retinal vessel segmentation method based on adaptive filter. Rodrigues and Marengoni, 2017 proposed a method based on morphology and wavelet transform, and Aguirre-Ramos et al. (2018) enhanced vascular contour through Gabor filter and Gaussian fractional derivative. Lu et al. (2016) used multi-scale filtering algorithm to preprocess images and Li et al., 2018 incorporated phase features to segment the retinal vessels. These traditional segmentation methods preliminarily achieved retinal vessel segmentation, but it still needs manual extraction of the image features due to the low accuracy in details, which may omit key details and fail to achieve end-to-end segmentation.

Deep learning based segmentation method developed rapidly in recent years. Yan et al. (2019) used a three-stage network model to segment the thin and thick vessels respectively, and then segmented the pixels by fusing the vessels. Liu (2021) proposed a method based on an optimized BP neural network, in which image features are extracted by adaptive histogram, matched filter and Hessian matrix. But the algorithm can only be applied on lesions in small area, and the large-scale lesion interference cannot be effectively avoided. Lu et al. (2021) segmented retinal vessels in fundus images through an attentional mechanism and conditional generative adversarial network, and Tang and Yu, 2021 adopted a BP neural network. In Reference (Dharmawan et al., 2019a), a hybrid algorithm was proposed by using a directional sensitive enhancement method and U-NET convolution network to train the enhanced image. But the algorithm is not optimized for multi-scale image segmentation, and the segmentation performance of vessels with lesions still needs improvement. These deep learning based methods have improved the accuracy of retinal vessel segmentation, but the segmentation performance still needs to be significantly improved in order to be widely used in machine-assisted health screening and identification in the future.

## 3 Network Structure

### Residual Module

To avoid the inevitable problems of deep neural networks as gradient disappearance and gradient explosion, residual learning (Chen et al., 2020; Feng et al., 2020; Yang et al., 2021) is introduced into the network. The parallel method is applied and the identity mapping is added to the output of the stacked convolution layer. It can effectively improve the feature extraction ability of the network. The function can be expressed as

**FIGURE 1:**
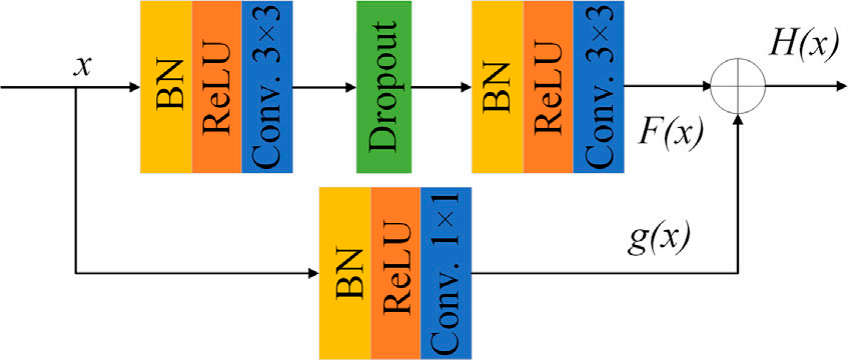
Schematic diagram of residual module.

F(x)=H(x)−x (1)

where *x* represents the input; *F*(*x*) represents the output of the jagged edge. If *F*(*x*) = 0, it becomes an identity mapping, while the input and output of the residual module are equal. *H*(*x*) represents the final output of the residual module. The structure of the proposed residual module is shown in Figure 1. Different from the ordinary residual module, the 1 × 1 convolution is added to the identity mapping to adjust the number of channels, whose function can be expressed as

H(x)=F(x)+g(x) (2)

Here *g*(*x*) represents the output of the convolution on the identity map.

The residual mapping of the residual module contains two 3 × 3 convolution layers and each convolution layer is processed by batch normalization (BN) (Awais et al., 2020) to accelerate the network convergence. Modified linear unit (ReLU) is used as the activation function, and L2 regularization is introduced to avoid network over-fitting. A dropout layer (Wang et al., 2019) (with random inactivation rate = 0.2) is added between the two stacked convolutional layers in the residual module to randomly discard some neurons during training, in order to prevent over-fitting and enhance generalization performance of the network.

### Atrous Spatial Pyramid Pooling

**FIGURE 2:**
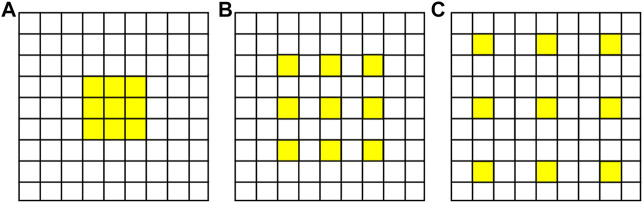
Schematic diagram of atrous convolution with different expansion rates as **(A)** *r* = 1, **(B)** *r* = 2, and **(C)** *r* = 3.

The receptive field of the convolution layer is related to the size of the convolution sum. Larger size means larger receptive field and stronger feature extraction capability of the network, but it also means more parameters to be trained. By utilizing atrous convolution, also called expansion convolution or extended convolution, firstly proposed by Chen et al., 2018, the receptive field can be enlarged without increasing the number of training parameters of the network. Compared with traditional convolution, atrous convolution introduced a hyperparameter called expansion rate *r*. The larger the value of *r* is, the larger the receptive field will be. When *r* = 1, the atrous convolution is equal to the ordinary convolution. Figure 2 is the schematic diagram of atrous convolution with different expansion rates.

**FIGURE 3:**
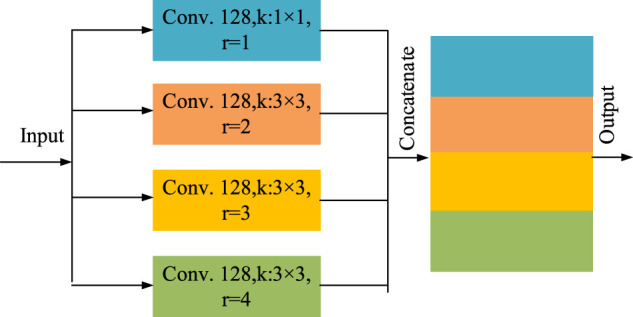
The structure diagram of the atrous spatial pyramid pooling.

In order to enlarge the receptive field of the network without adding too many parameters, so as to increase the feature extraction capability, we utilize the outputs of atrous convolution with different expansion rates and stack them to form the atrous spatial pyramid pooling (ASPP) (Li M. et al., 2021; Lian et al., 2021), The structure is shown as Figure 3.

This module is mainly composed of four parallel atrous convolution. including a 1 × 1 ordinary convolution (*r* = 1) and three 3 × 3 atrous convolution with expansion rate *r* = 2, 3 and 4 respectively. The number of convolution kernels of each convolution layers is set as 128. The outputs of the four convolution layers are concatenated as the total output of the ASPP. The multi-scale characteristic information of different receptive fields can be learned with different expansion rates, which can increase the recognition ability of small vessels. In addition, it also reduces the parameters that need to be trained and increases the training speed of the network.

### Residual Attention Module

With the rapid development of deep learning, it has become particularly important to add attention mechanism into the network in recent years. The attention mechanism of image recognition is mostly constructed by masks. Essentially, it is to train the weight of another layer to identify the key information in the image, so as to increase the sensitivity of the network to the key information. The network can be trained to notice the key areas of each image to generate attention. Spatial transformer network (STN) model (Xu et al., 2021b) uses the attention mechanism to transform the spatial information of the original picture into another space while retaining the key information. In the meantime, Reference (Hu et al., 2020) proposed a SENet model, whose core idea is to learn the weight of each channel through the attention module, in order to generate attention in the channel domain.

**FIGURE 4:**
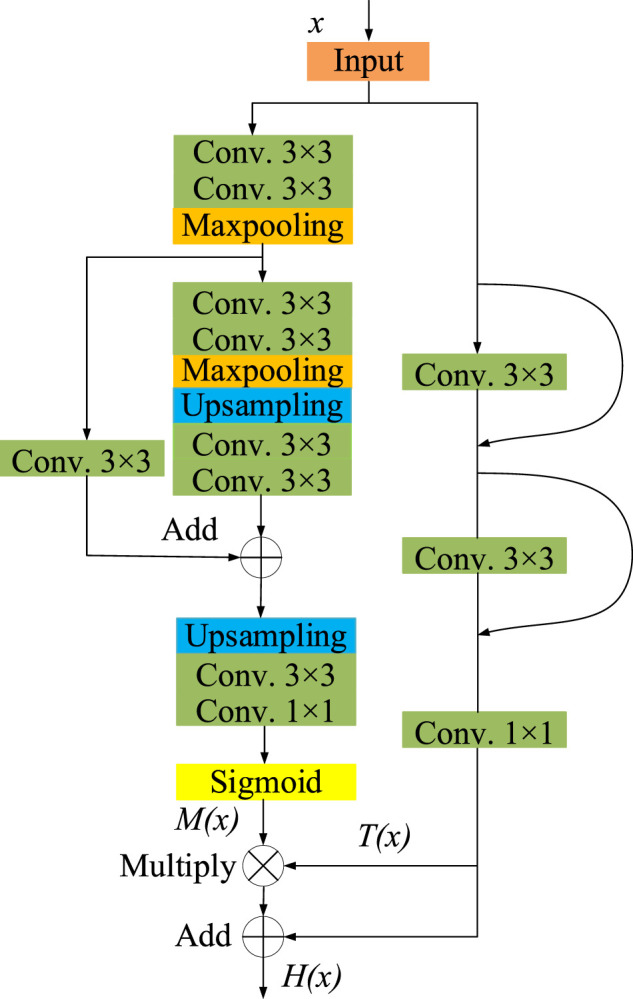
The schematic diagram of residual attention module.

In this paper, we propose a residual attention module, whose network structure is shown as Figure 4. This module is mainly composed of two parts, mask branch and trunk branch, whose output is represented by *M*(*x*) and *T*(*x*) respectively. Batch normalization and Rectified Linear Unit (ReLU) activation functions are used by default for all 3 × 3 convolutional layers. The trunk branch is composed of two 3 × 3 convolution layers and jump connections, which is used to extract feature information. In the mask branch, two down sampling and two up-sampling are carried out. After the process of Sigmoid activation function, a mask *M*(*x*) with the same size as the output of the trunk branch is obtained as the weight of the trunk output *T*(*x*). It can be expressed as

H(x)=(1+M(x))T(x) (3)

where *x* represents the input, *M*(*x*) represents the output of the mask branch, *T*(*x*) represents the output of the trunk branch, and *H*(*x*) represents the output of the residual attention module.

### Retinal Vessel Segmentation Network

**FIGURE 5:**
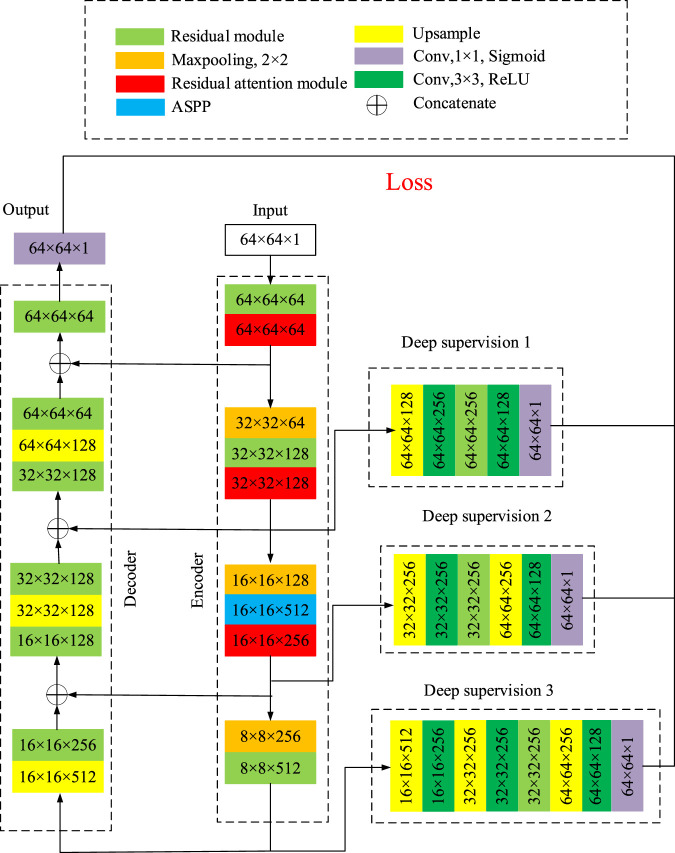
Structure diagram of the retinal vessel segmentation network.

The structure of the segmentation network proposed in this paper is shown as Figure 5, which is mainly composed of encoder, decoder and three deep supervision modules.

The large size of the input image may affect the segmentation, so it is necessary to cut apart the fundus image. The image is cut by windowing of 64 × 64 pixels, and the sliding step is set to be 16. The dataset can be expanded by randomly flipping, rotation and cropping the images. In Figure 5, the width and height of the input image of the network is 64, and the number of channels is 1, which is written as 64 × 64 × 1. During each step, the size of the image and the number of channels will change. The number represents the width × height × channel number, as shown in Figure 5. In the encoder network, the residual module and the ASPP, together with the residual attention module, will not change the width and height of the image, but increase the number of channels. The 2 × 2 maxpooling layer reduce the width and height of the image by half and keeps the number of channels constant. In the decoder network, up-sample block doubles the width and height of the image, and the residual module adjusts the number of channels. After each stage of up-sampling and residual modules, stack with the branches on the encoder. The dimension of the image after decoder network is 64 × 64×64. Finally, after the residual module and the convolution layer with kernel size of 1 × 1, the width and height of the image became 64 and the number of channels is 1. As a result, the dimension of the output image is 64 × 64 × 1.

Three branches are introduced in the encoder as the input of the three deep supervision modules with different layers and blocks. The output of the three deep supervision modules and the output of the decoder are used together to calculate the loss function and update the weight parameters. Up-sampling is carried out by means of transposed convolution, and a symmetric coding-decoding network is constructed by splicing the low-level features of encoder and high-level features of decoder with the method of jump connection. A 1 × 1 convolution is used at the last layer of the decoder to adjust the number of channels to 1. Finally, the Sigmoid activation function is used to scale the output to the range from 0 to 1, which can be expressed as

$$f\left(x\right)=\frac{1}{1+e^{-x}}$$ (4)

where *x* represents the input of the activation function, *f*(*x*) represents the output of the activation function, whose value is normalized.

The loss function of the network is composed of the loss generated by three depth supervision modules and the trunk network. The function can be written as

Loss=βLoss1+βLoss2+βLoss3+βLoss4 (5)

Here, *Loss* represents the total loss of the network, *Loss1*, *Loss2* and *Loss3* represent the loss generated by the three deep supervision modules respectively, and *Loss4* represents the loss generated by the output layer of the decoder. $\beta =1-\frac{\hat{epochs}}{epochs}$ , $\hat{epochs}$ represents the iteration times of the current network, and *epochs* represents the total iteration times of the network. It can be seen that as the iteration of the network approaches 0, the weight of loss β generated by the deep supervision module decreases gradually.

Since the pixel number of vascular and non-vascular may can be quite different, the binary cross entropy loss function with weight coefficient (Li X. et al., 2019; Jamin and Humeau-Heurtier, 2020) is adopted to reduce the uneven distribution of positive and negative samples, and its mathematical expression is defined as

$$Loss\left(\text{n}\right)=-1/m\sum_{i=1}^{m}\partial y_{i}\log\left(\hat{y_{i}}\right)+\left(1-\partial \right)\left(1-y_{i}\right)\log\left(1-\hat{y_{i}}\right),n=1,2,3,4$$ (6)

Here, *Loss*(*n*) represents the *Loss1*, *Loss2, Loss3 and Loss4* in Eq. 5, with *n* = 1,2,3,4 respectively; *m* represents the total number of pixels in the input image; *y*_*i*_ represents the label with value of 0 or 1, 0 represents background, and 1 represents blood vessel. $\hat{y_{i}}$ represents the output of the network, $\partial =X_{-}/m$ , $1-\partial =X_{+}/m$ , where $X_{-}$ and $X_{+}$ represent the number of non-vessel pixels and the number of vessel pixels respectively.

The batch size of the training is 20, the network iterates for 500 times, the learning rate is set at 0.001, and Adam is used as the optimizer.

## 4 Experiment and Analysis

### Database and Training Environment

The network model is established on deep learning framework based on TensorFlow. The hardware configuration of the experiment is i5-1100K and GTX1030, and the software runs on the Win10 system. The database used for the experiment includes Digital Retina Images for Vessel Extraction (DRIVE) (Staal et al., 2004) and Structured Analysis of the Retina (STARE) (Hoover et al., 2000). The DRIVE data set has 20 training images and 20 test images with the resolution of 584 × 584 pixels. The STARE data set contains 20 images with the resolution of 605 × 700 pixels, in which ten images are used as the training set and the other ten as the test set.

### Image Preprocessing

Environmental factors, such as illumination, interference and background, will affect the image segmentation, which leads to unsatisfactory result. Therefore, in order to further improve the accuracy of vessel segmentation, appropriate preprocessing operations are required for fundus images. Firstly, the image is transformed into grayscale image, and secondly, Gaussian filter is carried out to eliminate the noise. Thirdly, local histogram equalization (Dhal et al., 2020; Shi, 2021) and Gamma transformation are performed to adjust the contrast of the image.

#### 4.1.1 Channel Separation

**FIGURE 6:**
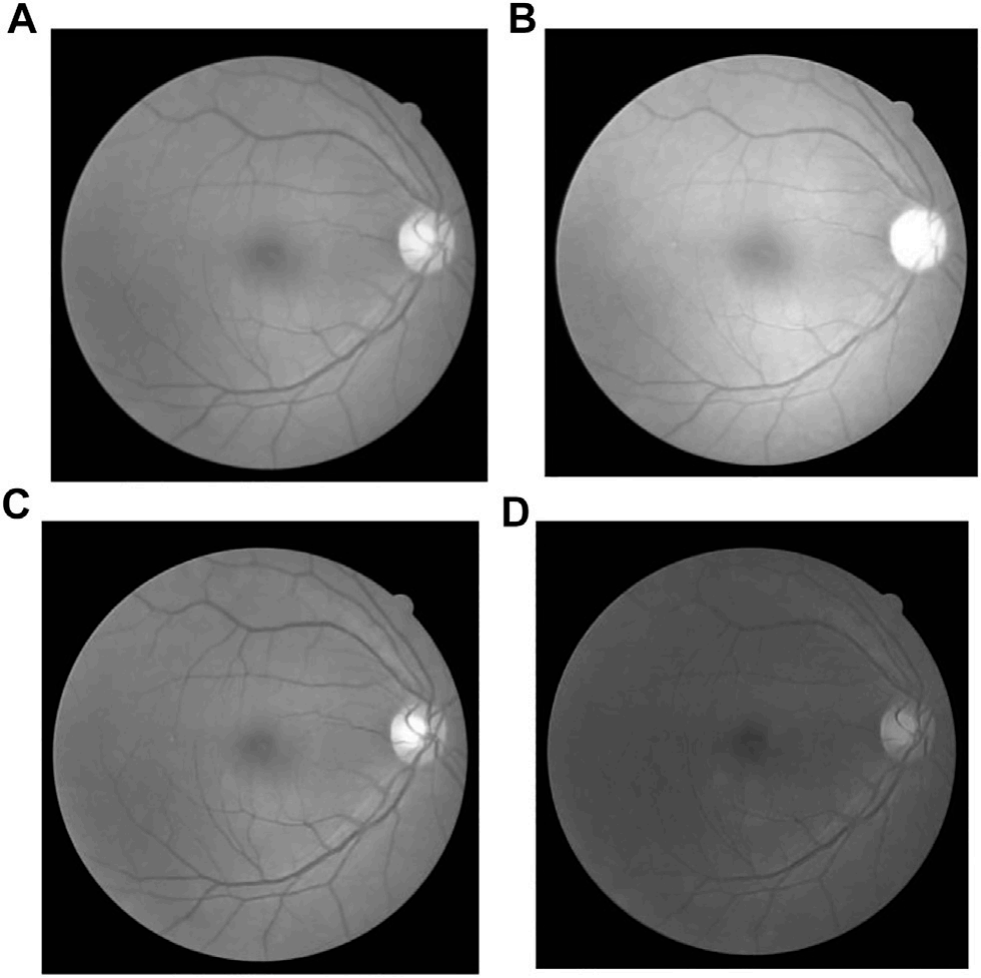
Original Image in different channels: **(A)** Original gray image, **(B)** Red channel image, **(C)** Green channel image, and **(D)** Blue channel image.

In the fundus examination, an RGB color image is obtained by the camera, composed of three separate channels: red, green and blue. The images are shown as Figure 6, in which (Figure 6A) is the original gray image, that is actually the average grayscale image of the three channels, and (Figures 6B,C,D are the original images in red, green, blue channel separately. The three-channel color image is too large and contains much useless data for subsequent processing. Single channel image information is enough for all the required information, which greatly reduce the amount of data processing and improve the computational efficiency. Channel compression and conversion is usually carried out by the weighted average of three images, or an optimal channel selection. To the human eye, the sensitivity to green is much higher than the other two colors (Ricci and Perfetti, 2007; Marin et al., 2011), as the contrast shown in Figure 6C. Therefore, the image in green channel is chosen instead of the original three-cannel image for subsequent image processing.

#### 4.1.2 Gray Histogram Equalization

Histogram (Li et al., 2020a; Sulewski 2020), also known as mass distribution map, is used for statistical reports, in which multiple bars with unequal heights are utilized to represent the distribution of data. During the image process here, the gray histogram represents the image distribution of gray level in the range [0, *L*-1]. The discrete function can be described as

$$p\left(r_{k}\right)=n_{k}/n$$ (7)

**FIGURE 7:**
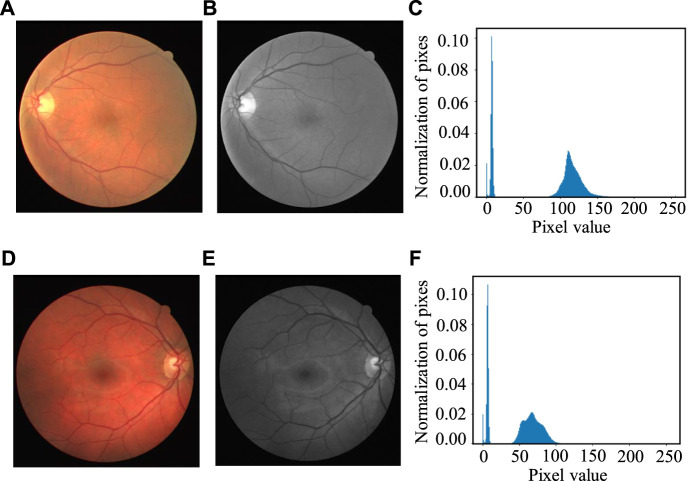
Two cases of gray histogram: **(A, D)** Original color image, **(B, E)** Gray image of the green channel and **(C, F)** Gray histogram of **(B, E).**

Here, *n* is the total number of images; *n*_*k*_ refers to the total number of pixels at the *k* ^*th*^ gray level; *r*_*k*_ refers to the *k* ^*th*^ gray level, and *k* = 0, 1, 2… *L*-1. In our experiment, *L* is set as 256. The gray histogram represents the frequency of pixels at each gray level shows in the image, with the gray level as the *x*-axis and the numbers of the pixels as the *y*-axis. Figure 7 shows two cases of the gray histogram of fundus images, with (Figures 7A,D) the original color images, (Figures 7B,E) the gray image after channel separation; (Figures 7C,F) the gray histogram of (Figures 7B,E) with the vertical axis as the normalized probability distribution of the pixels.

The goal of histogram equalization is to transform the original image from a certain concentration range of pixel values to a wider range so that the contrast between the similar gray value increase. The specific way is utilizing nonlinear stretch to transform the certain histogram to uniform distribution in a certain range, which can be described as

s=T(r), 0≤r≤L−1 (8)

Here, *r* represents the pixel gray value before the transformation, *s* represents the pixel gray value after the transformation, and *T*(*r*) represents the transformation function. *T*(*r*) is a monotone increasing function in the range of 0≤ *T*(*r*) ≤L-1, which ensures that the variables *r* and *s* are one-to-one corresponding. 0≤ *r* ≤*L*-1 and 0 ≤ *T*(*r*) ≤ *L*-1 can ensure the range of gray value after transformation will not exceed the original one.

We use *p*_*r*_(*r*) and *p*_*s*_(*s*) to represent the probability density function corresponding to gray level *r* and *s* respectively. The inverse transformation from *s* to *r* can be described as

$$r=T^{-1}\left(s\right),\;\;0\leq s\leq L-1$$ (9)

The inverse transformation function *T* ^-1^(*s*) also meets the monotonically increasing condition in the range of 0 ≤ *s* ≤ *L*-1. According to the theory, if *p*_*r*_(*r*) and *T*(*r*) are known and *r* = *T* ^-1^(*s*) is a monotonically increasing function, the gray level probability density function *p*_*s*_(*s*) of the image after transformation is shown as follows:

$$p_{s}\left(s\right)=p_{r}\left(r\right)\left|\frac{dr}{ds}\right|$$ (10)

The transformation function of the histogram equalization function can be written as

$$s=T\left(r\right)=\left(L-1\right)\int_{0}^{r}p_{r}\left(w\right)dw$$ (11)

Here, *w* is the integral variable. It can be seen from the right side of the Eq. 11 that the integral is the area below the function curve, so it meets the condition of monotone increasing. As the integral of *p*_*r*_(*r*), the probability density function of *r*, on the range [0, *L*-1] is 1, and *s* takes the maximum value *L*-1, which does not exceed the gray range of *r*, we can get the following formula as

$$\frac{ds}{dr}=\frac{dT\left(r\right)}{dr}\left(L-1\right)\frac{d}{dr}\left[\int_{0}^{r}p_{r}\left(w\right)dw\right]=\left(L-1\right)p_{r}\left(r\right)$$ (12)

Substitute Eq. 12 into Eq. 10, the probability density function *p*_*s*_(*s*) can be written as

$$p_{s}\left(s\right)=p_{r}\left(r\right)\left|\frac{dr}{ds}\right|=p_{r}\left(r\right)\left|\frac{1}{\left(L-1\right)p_{r}\left(r\right)}\right|=\left|\frac{1}{L-1}\right|,\text{0}\leq s\leq L-1$$ (13)

Through the above equation, *p*_*s*_(*s*) is proved uniformly distributed, which indicates that we can obtain a gray image with more uniform gray distribution and higher contrast by histogram equalization transformation.

For digital images, the gray level is discrete, and the sum of the probability tensity function should be used instead of the integral. The probability of occurrence of gray level *r*_*k*_ is approximately transformed into

$$p_{k}\left(r_{k}\right)=\frac{n_{k}}{n},\;\;\;k=0,1,\ldots ,L-1$$ (14)

Here, *n* is the number of all pixels in the image, *n*_*k*_ is the number of pixels on the gray-level *r*_*k*_, *L* is the total number of possible gray-level in the image, so the discrete form of the transformation function is

$$s_{k}=T\left(r_{k}\right)=\left(L-1\right)\sum_{j=0}^{k}\frac{n_{j}}{n}\;\;\;k=0,1,\ldots ,L-1$$ (15)

The operation of discrete histogram equalization is to map each pixel in the input image of gray-level *r*_*k*_ to the pixel in the output image of gray-level *s*_*k*_ through Eq. 15. Different from the continuous form, the new image generated is not necessarily completely evenly distributed, but the image tends to be uniform as well with a higher contrast and a larger grayscale after transformation.

**FIGURE 8:**
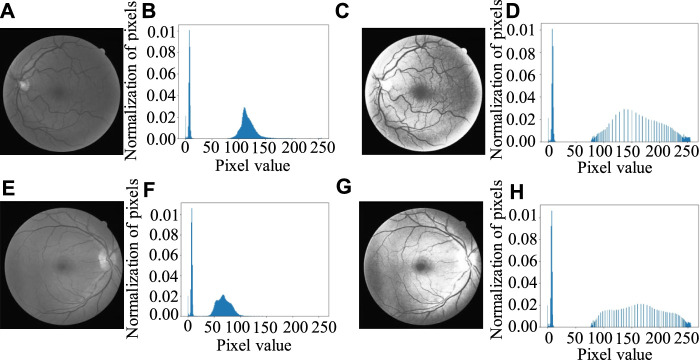
Histogram equalization results: **(A, E)** Gray image of green channel, **(B, F)** Original gray histogram of **(A, E)** separately, **(C**, **G)** Image after local histogram equalization, **(D, H)** Gray histogram after local histogram equalization.

For the fundus images, the background will affect the histogram equalization, so the local histogram equalization (Lai et al., 2015) transformation for the eyeball part is used instead of global histogram equalization. Figure 8 shows the result of local histogram equalization. It can be seen from Figure 8 that the value of background pixels does not change as close to 0, while the histogram of the eyeball part is obviously stretched and the contrast improves obviously.

#### 4.1.3 Gamma Transformation

Gamma transformation (Hoo et al., 2017; Wang et al., 2018; Wang et al., 2021) can be used to adjust the contrast of gray images which are overexposed or underexposed. Nonlinear transformation is utilized to enhance the gray value of the dark area and reduce the gray value of the overexposed area, so that the overall detail of the image will be enhanced. The formula of Gamma transformation can be written as

$$s=cr^{\text{γ}}$$ (16)

**FIGURE 9:**
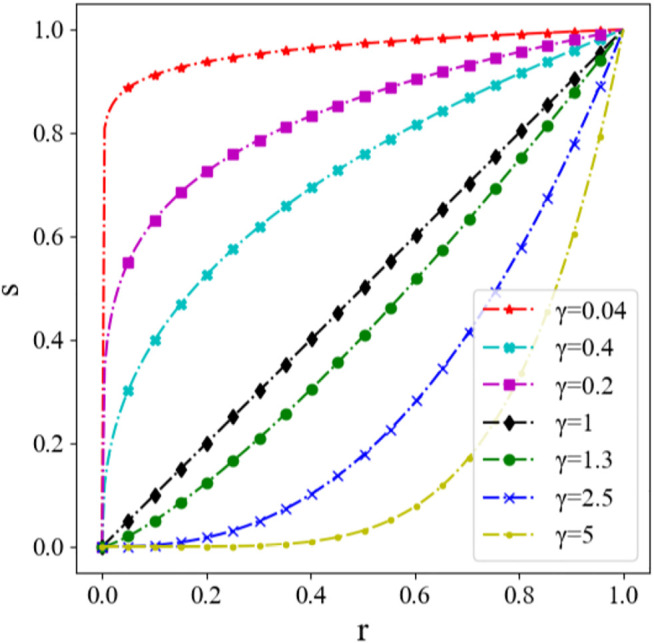
Schematic diagram of Gamma transformation with different factor γ.

Here, *r* is the input value of the gray image, with the range of [0,1]; *s* is the output value after Gamma transformation; *c* is the gray scale coefficient, usually equals to 1; *γ* is the Gamma factor, which controls the stretch of the entire transformation. The transformation with different Gamma factor *γ* is shown in Figure 9, where the abscissa and the ordinate represent the gray value before and after Gamma transformation for a certain pixel individually. It can be seen that the result differs with the factor *γ*.

**FIGURE 10:**
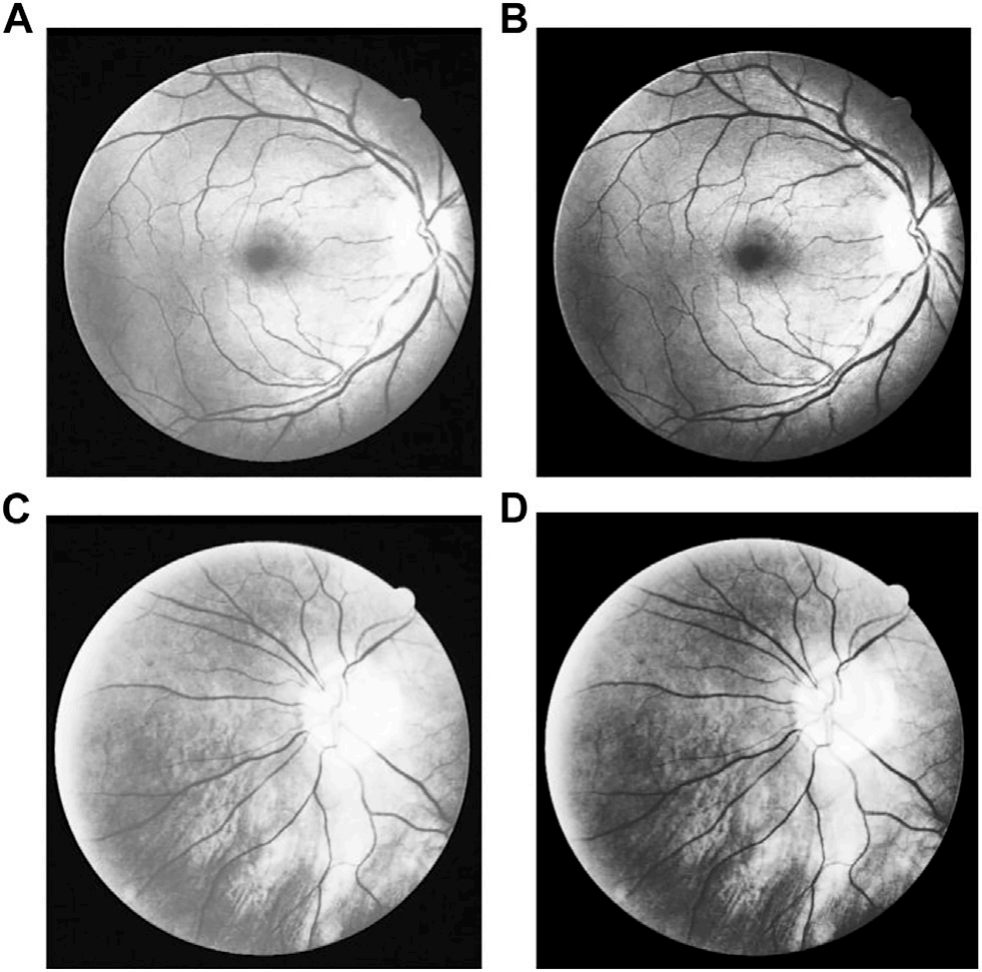
Result of Gamma transform: **(A, C)** Images after histogram equalization, **(B, D)** Images after Gamma transform of **(A, C)** separately.

Gamma transformation is used to process the histogram equalization image, in order to further enhance the contrast. It is drawn from the experiment that the best contrast enhancement effect is with *γ* = 1.3. The Gamma transformation results with *γ* = 1.3 are shown in Figure 10.

#### 4.1.4 Training Data Preparation

**FIGURE 11:**
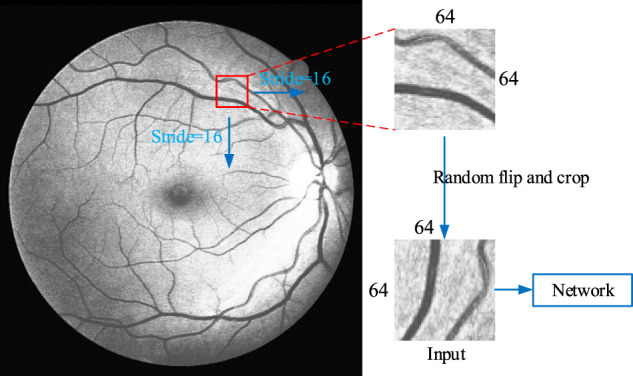
Schematic diagram of windowing and data preparation.

As the pixel number of fundus images collected by different instruments is not the same, and also the size of the whole image is relatively large, the segmentation accuracy will be affected and small retinal vessels that contains vital information cannot be extracted when using the full-size images as the direct training data. Therefore, we adopt a sliding window on the image to capture certain areas as input, as shown in Figure 11. Firstly, fill the background pixel with the value of 0 to widen the height and the width of the image as integer multiples of 64. Secondly, a 64 × 64 window is used to slide from the upper left corner of the image, from left to right and top to bottom, with a sliding step as 16. The sliding window of 64 × 64 is fixed in this network, but the sliding step size can be change. The smaller the sliding step size is, the more data can be obtained, and larger slide step means less data obtained. The maximum sliding step size should not be greater than 64, as greater than 64 results in some aeras with information will be missed. In the meantime, the sliding step should not be too small, in order not to get a lot of overlapping areas. The sliding step is set as 16, as a total of 27380 64 × 64 training data can be obtained from DRAVE data set, and 15170 64 × 64 training data from STARE data set. The obtained data are randomly flipped and clipped in each epoch to ensure that the training data we sent to the network was different in each epoch.

### Evaluation Indexes of Model Performance

Retinal vessel segmentation is to classify pixels and determine whether each pixel belongs to blood vessels. Four evaluation methods are used to evaluate the effect of vessel segmentation, which are accuracy *R*_*Acc*_, sensitivity *R*_*Se*_, specificity *R*_*Sp*_ and ROC curve. The function of the former three are as follows:

$$R_{Acc}=\frac{T_{P}+T_{N}}{T_{P}+T_{N}+F_{P}+F_{N}}$$ (17)

$$R_{Se}=\frac{T_{P}}{T_{P}+F_{N}}$$ (18)

$$R_{Sp}=\frac{T_{N}}{T_{N}+F_{P}}$$ (19)

Here *T*_*P*_ represents true positive, whose value equals to the number of accurately segmented vessel pixels; *T*_*N*_ represents the true negative, and its value equals to the number of correctly segmented background pixels; *F*_*P*_ represents false positive, with the value equals to the number of incorrectly segmented vessel pixels; *F*_*N*_ represents the false negative, and its value equals to the number of wrongly segmented background pixels. A certain curve can be draw by the false positive rate (1-*R*_*Sp*_) as the horizontal coordinate and the true positive rate (*R*_*Se*_) as the vertical coordinate, which is the ROC curve (Hoo et al., 2017; Michael et al., 2019). The area under the curve is defined as *R*_*AUC*_ (Janssens and Martens, 2020; Muschelli, 2020), and the closer its value to 1, the better the segmentation effect is.

### Experimental Results

**FIGURE 12:**
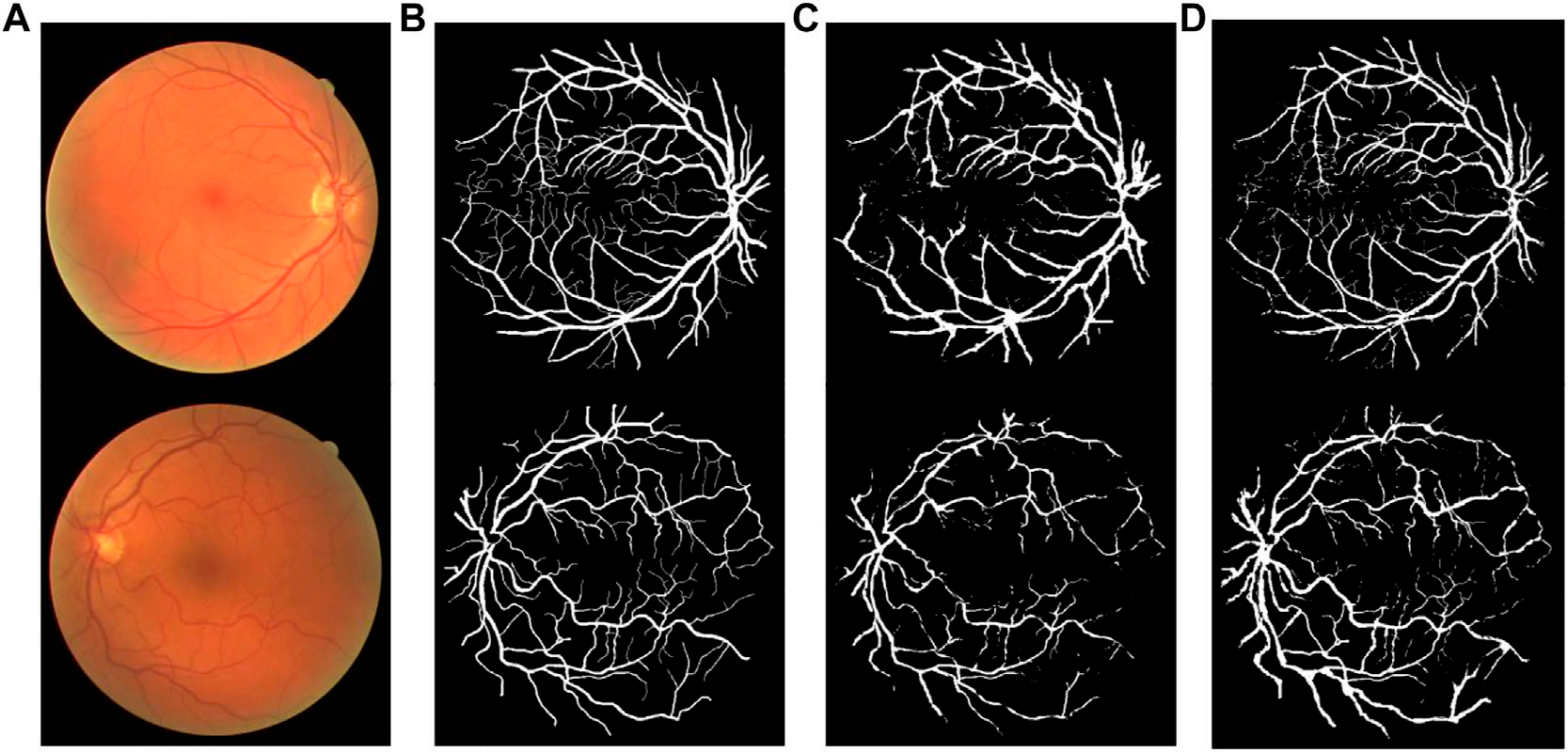
Comparison of segmentation results of different algorithms on DRIVE data sets: **(A)** Original fundus image, **(B)** Reference standard, **(C)** Results in Ref. (Fan et al., 2019), and **(D)** Results by proposed method.

**FIGURE 13:**
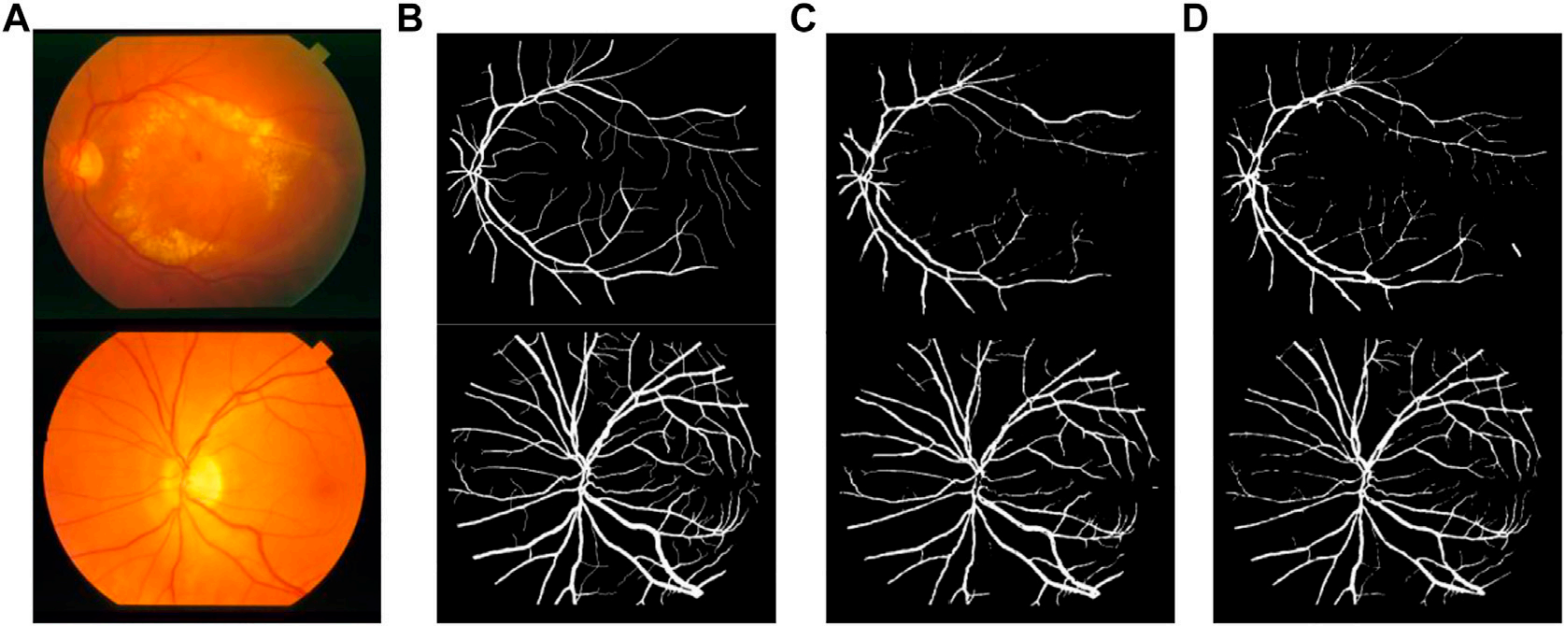
Comparison of segmentation results of different algorithms on STARE data sets: **(A)** Original fundus image, **(B)** Reference standard, **(C)** Results in Ref. (Fan et al., 2019), and **(D)** Results by proposed method.

Figures 12, 13 show the segmentation result on the DRIVE data set and STARE data set individually, in which (Figures 12A, 13A) is the original fundus image; (Figures 12B, 13B) is the image of retinal vessel manually segmented by experts, which is used as the standard reference; (Figures 12C, 13C) is the segmentation result of the typical traditional method in Reference (Fan et al., 2019); and (Figures 12D, 13D) is the segmentation result based on the proposed method. It can be observed from Figures 12, 13 that the segmentation image of the network is mostly consistent with the expert labeled image, and the capillaries are much more detailed than the traditional method. The vast majority of characteristic information for retinopathy recognition and identification is contained in these details.

**FIGURE 14:**
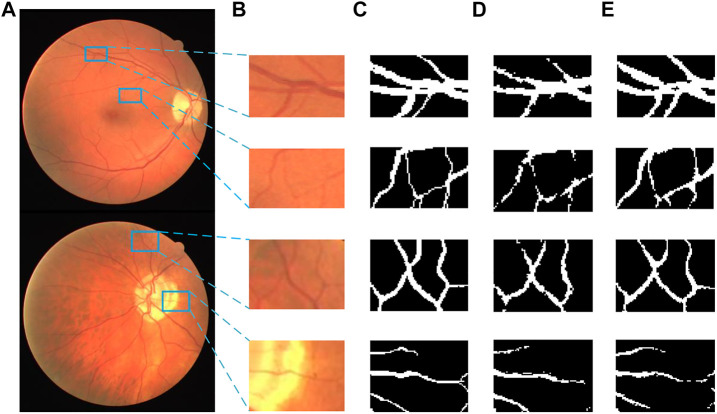
Comparation of local segmentation effects on DRIVE data sets: **(A)** Original fundus image; **(B)** Original local image; **(C)** Local reference standard image; **(D)** Local segmentation results in Ref. (Fan et al., 2019); and **(E)** Local results by proposed method.

**FIGURE 15:**
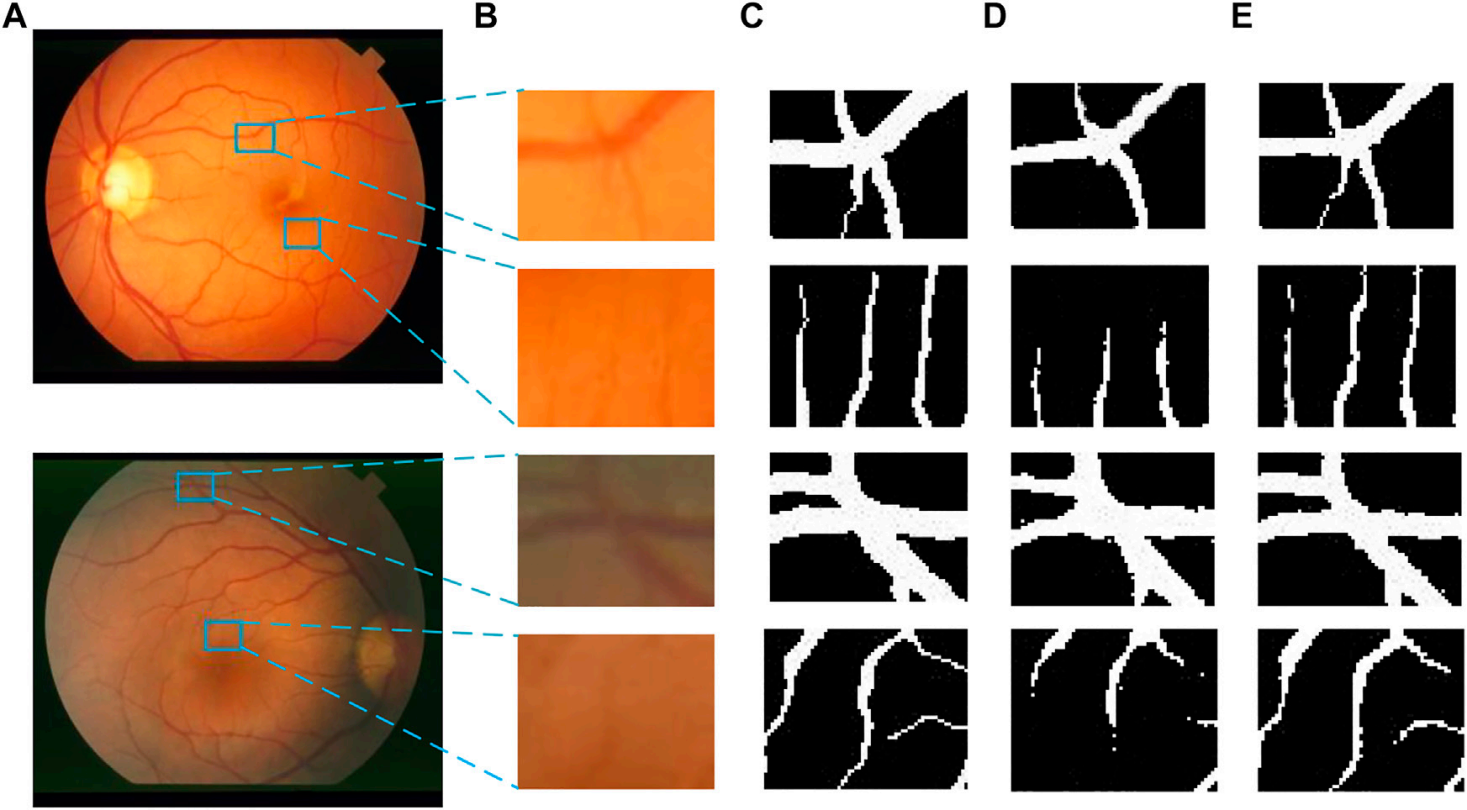
Comparation of local segmentation effects on STARE data sets: **(A)** Original fundus image, **(B)** Original local image, **(C)** Local reference standard image, **(D)** Local segmentation results in Ref. (Fan et al., 2019), and **(E)** Local results by proposed method.

Figures 14, 15 show the segmentation results of some local areas on the DRIVE data set and STARE data set separately, in which (Figures 14A, 15A) is the original fundus image with the selected local areas by the blue boxes; (Figures 14B, 15B) is the original image of selected areas; (Figures 14C, 15C) is the reference standard images of the selected areas; (Figures 14D, 15D) is the segmentation result based on method in Reference (Fan et al., 2019); and (Figures 14E, 15E) is the result based on the proposed method. The chosen local areas include relatively thick blood vessels, intersecting blood vessels, and thin blood vessels. It can be observed from Figures 14, 15 that the vessel pixels division has a higher accuracy in both thick and thin vessels, resulting in better continuity of blood vessels and fewer broken vessels, which is more conducive to extract key feature information.

We compare the segmentation performance of the proposed algorithm and the methods in the references, applied on DRIVE data set and STARE data set, as shown in Tables 1, 2 respectively. The accuracy of the proposed method on DRIVE data set and STARE data set is 0.9590 and 0.9688 respectively. The sensitivity is 0.8320 and 0.8432, while the specificity is 0.9885 and 0.9785 severally. The accuracy, sensitivity, and specificity on both data sets are generally improved compared to other algorithms. The ROC curve index of the algorithm in this paper is 0.9786 and 0.9820 individually. Though the *R*_*AUC*_ is 0.0073 lower than the latest Reference (Lu et al., 2021), the specificity is significantly improved by the algorithm proposed in this paper, and the overall performance is improved in return. As for the STARE data set, the sensitivity of the proposed algorithm is 0.0137 lower than that in Reference (Dharmawan et al., 2019b), but all the other evaluation indicators improved. Therefore, the segmentation performance of the proposed method is effectively improved.

**TABLE 1:** Segmentation performance comparison of different algorithms on DRIVE.

Method	*R*_*Acc*_	*R*_*Se*_	*R*_*Sp*_	*R*_*AUC*_	Years
Rodrigues et al.	0.9465	0.7165	0.9801	-	2017
Li et al.	0.9574	-	-	0.9702	2018
Aguirre-Ramos et al.	0.9503	0.7854	0.9662	-	2018
Girard et al.	0.9480	0.7490	0.9770	-	2019
Fan et al.	0.9531	0.7035	0.9763	-	2019
Li Z Q et al.	0.9375	0.6945	0.9729	0.9320	2020
Pachade et al.	0.9405	0.7514	0.9676	-	2020
Lu et al.	0.9559	0.8288	0.9745	0.9786	2021
Liu	0.9582	0.8137	0.9055	-	2021
Proposed method	**0.9590**	0.8320	0.9885	0.9713	2021

R_Acc_ represents the accuracy; R_Se_ represents the sensitivity; R_Sp_ represents the specificity; R_AUC_ represents the area under the ROC curve.

**TABLE 2:** Segmentation performance comparison of different algorithms on STARE.

Method	*R*_*Acc*_	*R*_*Se*_	*R*_*Sp*_	*R*_*AUC*_	Years
Li Z Q et al.	0.9461	0.7456	0.9693	0.9524	2020
Pachade et al.	0.9543	0.7769	0.9688	-	2020
Lu et al.	0.9683	**0.8432**	0.9775	0.9813	2021
Proposed method	**0.9688**	0.8295	**0.9785**	**0.9820**	2021

R_Acc_ represents the accuracy; R_Se_ represents the sensitivity; R_Sp_ represents the specificity; R_AUC_ represents the area under the ROC curve.

**FIGURE 16:**
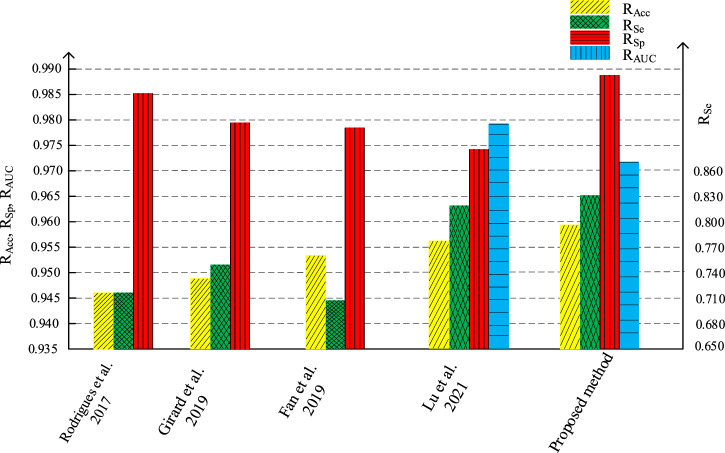
Comparations of evaluation indicators with different algorithm on DRIVE.

**FIGURE 17:**
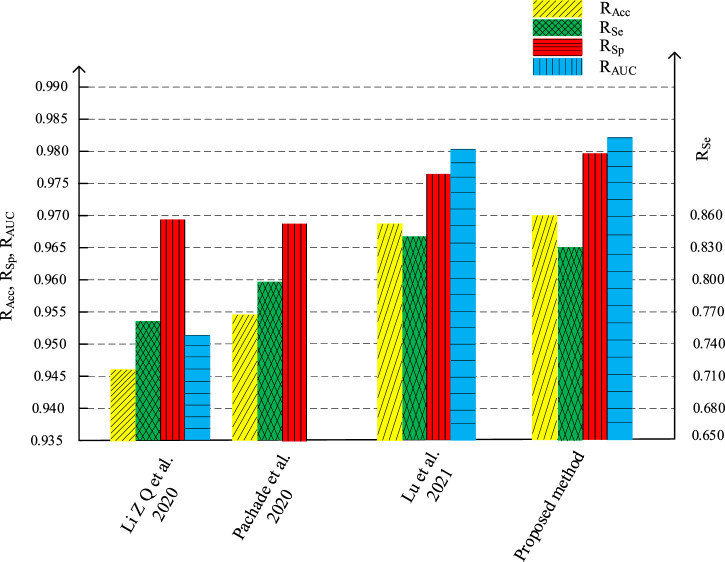
Comparations of evaluation indicators with different algorithm on STARE.

Figures 16, 17 are the histogram of the evaluation indexes of the proposed algorithm and other typical algorithms in DRIVE data set and STARE data set respectively. It can be seen more intuitively from the figure that the proposed algorithm has better segmentation performance and smaller error than other algorithms.

## 5 Conclusion and Future Work

The proposed residual convolution neural network based retinal vessel segmentation algorithm has been proved as an effective way to extract the blood vessel from the fundus images. The accuracy of the proposed method on DRIVE data set and STARE data set reaches 0.9590 and 0.9688 respectively, with the sensitivity and specificity on both data sets generally improved compared to other algorithms. It has a better segmentation performance and smaller error than the existing methods, especially in the details of capillaries, which contains most of the key feature information of diagnose and identification. In addition, by introducing the ASPP module, the receptive field is enlarged and the number of training parameters is reduced, which means a great potential for sharply increasing the volume of the identifications data and shortening the recognition time.

On the basis of this work, it is still necessary to do further research on obtaining more detailed capillary features, and further extract features from the fundus image data of different pathologic conditions, different disease courses, or different healthy people. Besides, specific criteria are needed to evaluate the details of the segmentation quantitively. Continuous research will provide more forceful support for the realization of computer-assisted retinal disease screening and retina identification in the future.

In addition, the network structure and construction method proposed in this paper are of great reference significance to many other applications. Especially the proposed improved residual attention module combined with deep supervision module successfully overcome the gradient disappearance and explosion in the convolution neural network. The encoder-decoder network structure effectively avoids inefficient learning and sharing in training, and the atrous spatial pyramid pooling significantly enlarges the receptive field while reducing the number of training parameters. These contributions have potential implications for other applications of biological heuristic algorithms, not limited to image processing problem (Tao et al., 2021b), but can even be applied in public opinion dissemination (Chen et al., 2021b; Chen et al., 2021c) and behavior analysis (Xu et al., 2021a; Xiang et al., 2021). Further extended research will provide broader support for future applications in other aspects.

## Data Availability

The original contributions presented in the study are included in the article/Supplementary Material, further inquiries can be directed to the corresponding author.
